# Impact of the COVID-19 Pandemic on Persons Living with HIV in Western Washington: Examining Lived Experiences of Social Distancing Stress, Personal Buffers, and Mental Health

**DOI:** 10.1007/s10461-024-04273-7

**Published:** 2024-03-16

**Authors:** Sarah Smith, Kristin Beima-Sofie, Asad Naveed, Nikki Bhatia, Murugi Micheni, Anh Tuyet Nguyen, Francis Slaughter, Liying Wang, Sandeep Prabhu, Stephaun Wallace, Jane Simoni, Susan M. Graham

**Affiliations:** 1 University of California, San Diego, CA USA; 2 University of Washington, Seattle, WA USA; 3 University of Toronto, Toronto, ON Canada; 4 National AIDS Control Council (NACC), Nairobi, Kenya; 5 Department of Medicine, Brigham & Women’s Hospital, Boston, MA USA; 6 Vaccine and Infectious Disease Division, Fred Hutchinson Cancer Research Center, Seattle, WA USA

**Keywords:** COVID-19 pandemic, HIV, Mental health, Coping

## Abstract

Pandemic-related stressors may disproportionately affect the mental health of people with HIV (PWH). Stratified, purposive sampling was used to recruit 24 PWH who participated in a quantitative survey on COVID-19 experiences for in-depth interviews (IDIs). IDIs were conducted by Zoom, audio recorded and transcribed. Thematic analysis was used to develop an adapted stress-coping model. Participants experienced acute stress following exposure events and symptoms compatible with COVID-19. Social isolation and job loss were longer-term stressors. While adaptive coping strategies helped promote mental health, participants who experienced multiple stressors simultaneously often felt overwhelmed and engaged in maladaptive coping behaviors. Healthcare providers were important sources of social support and provided continuity in care and referrals to mental health and social services. Understanding how PWH experienced stressors and coped during the COVID-19 pandemic can help healthcare providers connect with patients during future public health emergencies, address mental health needs and support adaptive coping strategies.

**Supplementary Information:**

The online version contains supplementary material available at 10.1007/s10461-024-04273-7.

## Introduction

The first case of COVID-19 in the United States was reported in Western Washington in January 2020 [1]. The World Health Organization declared COVID-19 a global pandemic in March 2020 and shortly thereafter, Washington State implemented a wide range of social distancing measures [1, 2]. The COVID-19 pandemic has since laid bare social and health inequities throughout the United States, including in Washington State. Numerous published reports demonstrate how COVID-19 disproportionately impacted vulnerable populations such as racial and ethnic minorities, individuals experiencing homelessness, persons who inject drugs, and other marginalized groups [3–6]. These communities are also vulnerable to and disproportionately impacted by HIV [7–9].

Understanding the psychosocial impacts of the pandemic for people living with HIV (PWH) is critical, as they are already at increased risk for common mental health disorders such as depression and anxiety [10–12], and poor mental health is often a predictor of negative HIV-related outcomes [13]. At the same time, a growing body of literature supports an increased susceptibility of people with pre-existing mental health conditions to stressors associated with COVID-19, relative to the general population [14].

The theory of stress and coping described by Lazarus and Folkman provides a powerful and useful framework for understanding how individuals cognitively appraise potentially stressful demands or events and utilize both internal and external resources to manage these stressors [15]. Multiple stressors, such as adhering to treatment regimens, disclosing HIV status, and managing HIV-related symptoms, have been shown to impact the mental health of PWH [10–12]. Stress and coping models guided by the Lazarus and Folkman theory [15], have been used to better understand PWH coping strategies and to develop and test interventions related to coping [16–18]. These studies have shown that effective coping strategies used by PWH include problem-focused coping, addressing the stressor directly, and emotion-focused coping techniques such as positive reappraisal, meditation and exercise to alleviate stress and improve mood [16–18]. Findings related to the effectiveness of social support in its various forms remain mixed [16, 17]. The Lazarus and Folkman framework [15] informed the current study’s aims to examine pandemic-related stressors, adaptive and maladaptive coping strategies, and mental health related outcomes among PWH.

Qualitative methods provide powerful analytical approaches that complement epidemiological studies, and may offer important, contextual insights regarding mental health and other determinants of clinical outcomes [19]. And yet, according to a scoping review published in May 2021, only 4.5% of research investigating the impacts of COVID-19 on PWH within the first 12 months of the pandemic were focused on stress and mental health, and less than 8% involved qualitative research [12]. A deeper understanding of how PWH made meaning of, responded to, and coped with the COVID-19 pandemic will help clinicians examine the ways in which adaptive and maladaptive coping strategies may have impacted care engagement and clinical outcomes. We conducted a qualitative study to explore the lived experiences of PWH in Western Washington, in order to better understand how the pandemic impacted daily life, identify resources that led to or mitigated symptoms of anxiety and depression, and develop an adapted stress-coping model of how COVID-19-related stress impacted mental health in this population.

## Methods

### Study Design and Population

This qualitative research study evaluates data collected as part of the University of Washington (UW) HIV and COVID Study, a mixed-methods assessment of COVID-19 impacts on PWH in Western Washington. The study included a cross-sectional, online survey in REDCap to evaluate experiences with and impacts of the COVID-19 pandemic and related social distancing on a range of health outcomes, as well as interviews with a purposively selected subset of survey participants. Individuals were eligible to participate in the survey if they were 18 or older, had internet access, and had enrolled in the UW HIV patient registry, which recruits UW clinic patients to participate in the Centers for AIDS Research Network of Integrated Clinical Systems (CNICS) cohort, a longitudinal, observational cohort of PWH in care at eight geographically distinct HIV research sites in the US from January 1995 to the present (http://www.uab.edu/cnics/) [20]. The HIV patient registry in Seattle includes patients who are 84% male and 67% white, with a median age of 52 (range 18–89). Survey recruitment was done via email and text message. After consenting, participants were asked a series of questions related to sociodemographic characteristics, COVID-19 impact, personal buffers, mental health, substance use, sexual health, and HIV treatment outcomes. Individuals who participated in the survey were asked if they were willing to participate in an in-depth interview (IDI). Participants who completed the survey by November 6, 2020, and who consented to participate in an IDI, were eligible to participate in this qualitative study.

In the survey, we included a seven-item COVID stress scale created by selecting items from the longer 36-item COVID Stress Scale to capture several dimensions of COVID-19 stress while minimizing participant burden [21]. This brief scale assessed different types of stress due to the pandemic (e.g., “I’m afraid of getting COVID-19”) and related social distancing measures (e.g., “Social distancing has resulted in increased mental stress”). For each item, participants indicated the intensity of the stress using a five-point Likert scale, ranging from 0 (not at all) to 4 (extremely). Responses to the seven items were summed to derive a total COVID-19 stress score, ranging from 0 (no impact) to 28 (high impact). Participants were then categorized as belonging to one of three tertiles: “Low Stress” (2–11), “Medium Stress” (12–16) or “High Stress” (17–24). As of October 31, 2020, 327 participants had completed the survey and 277 (85%) had consented to IDI invitation: 103 participants scored as having “Low Stress”, 95 as “Medium Stress”, and 79 as “High Stress.” To capture a wide range of experiences, and to better examine factors that led to or mitigated symptoms of anxiety and depression, we used stratified, purposive sampling to select participants from this population who scored in the “Low Stress” and “High Stress” categories, aiming for representative coverage of the sample by age, sex, race/ethnicity, and sexual orientation.

### Data Collection

The HIV and COVID study was guided by the Lazarus and Folkman theory of stress and coping [15]. Our initial model focused on how stressors resulting from the COVID-19 pandemic (e.g., COVID-19 illness, social distancing stress, job loss, housing challenges, reduced quality of life) may have been mitigated by adaptive coping. We were interested in how personal buffers (i.e., coping resources) such as COVID-19 knowledge, support for social distancing, technology access and skills, social support, and prior experience with coping strategies would impact health outcomes such as mental health, substance use, sexual health, and HIV treatment adherence. The stress-coping model applied in this qualitative analysis posited that each individual’s COVID-19 experience was the result of a stressful/traumatic life event (i.e., the COVID-19 pandemic), and that personal buffers were protective factors buffering the negative effects of COVID-19 on health outcomes.

Based on this model, we developed a four-part semi-structured interview guide that captured: (1) participants’ knowledge of COVID-19, (2) their personal experiences and beliefs about COVID-19, (3) how social distancing measures influenced daily life, and (4) how the pandemic impacted their motivation and ability to access health care and other services, including those related to mental health and substance use treatment.

IDIs were conducted by SS and MM between December 2020 and May 2021 using HIPAA compliant online Zoom or phone-to Zoom. Participants were recruited for IDIs via email. All IDIs were conducted in English, and were audio recorded via Zoom with participants’ consent. Participants who completed an interview received $40 for their time. After the first 5 interviews, the interview guide was revised to optimize phrasing and question flow, and several probes were added, in order to better capture participant experiences and perspectives (Supplemental File 1). The average length of IDIs was 55 min, ranging from 35 min to 1 h and 33 min.

Following each IDI, a structured debrief report was written to capture observations and summarize participant responses. Otter.com AI software was used to provide first-draft transcriptions of the recorded interviews. Draft transcripts were reviewed and revised by SS or NB to ensure accuracy and quality.

The study protocol, consent form, and IDI topic guide were reviewed and approved by the UW Human Subjects Division. All study participants provided written informed consent, with oral confirmation upon starting the interview.

### Data Analysis

We conducted thematic analysis [22], grounded in our adapted COVID-19 stress-coping model, to identify pandemic-related stressors, personal buffers that acted as protective factors mitigating negative effects, and reflections on personal mental health. All transcripts were coded using the qualitative analysis software ATLAS.ti (ATLAS.ti version 8, Scientific Software Development GmbH, Berlin, 2020). A preliminary codebook was developed deductively based on the domains of our conceptual model, then expanded through open coding and identification of data-driven codes. During three rounds of consensus coding, discrepancies in code application were resolved through discussion with the larger analysis team, until a final codebook and coding strategy were agreed upon. Thereafter, the remaining transcripts were divided between two analysts (SS and AN) and imported into ATLAS.ti for independent coding. After all transcripts were independently coded, both analysts reviewed each other’s codes and text segmentation, and resolved disagreements [22].

Salient themes were identified through generating and reviewing queries of codes within each conceptual area (stressors, personal buffers, and mental health outcomes) [22]. Interpretation of findings were discussed among the analysts and by the broader study team. Finally, a data display was developed to include illustrative quotes corresponding to stressors, personal buffers, and coping strategies [22].

## Results

Of the 59 participants invited to interview, 24 (41%) completed an interview: 11 were individuals who had low COVID stress scores and 13 were individuals who had high COVID stress scores. Table 1 presents characteristics of these participants. Median age was 48 years (range 28–67 years), and 18 (75%) identified as male. Sixteen (67%) identified as white, and 16 (67%) identified as gay, homosexual or lesbian. Due to our purposive sampling approach, these demographics closely matched those of the overall sample who participated in the online survey (median age 47, 82% male, 65% white, and 66% gay, homosexual or lesbian). The majority of participants (80%) had at least some college, an associate’s degree, or technical school training. Notably, while many lived in low-income housing, no participants experienced homelessness during the early months of the pandemic, although two younger participants reported fear and/or loss of housing. Both PHQ-8 and GAD-7 scores were higher among participants with high COVID stress scores, compared to those with low scores.

**Table 1:** Sociodemographic characteristics of participants, by stress category and overall

Continuous variables	Overall sample (n = 24)	Low stress (n = 11)	High stress (n = 13)	Comparison	
	Median (IQR)	Median (IQR)	Median (IQR)	Wilcoxon rank-sum	
				Z value	P value
Age in years	48 (36–58)	45 (32–58)	52 (38–58)	0.319	0.75
PHQ-8	7.5 (5–10.5)	5 (2–10)	10 (7–14)	2.479	0.01
GAD-7	7 (1.5–10)	2 (0–7)	9 (7–14)	2.796	0.005

Categorical variables	N (%)	N (%)	N (%)	Fisher exact	
				Test statistic	P value
Gender identification				N/A	0.80
Male	18 (75.0)	8 (72.7)	10 (77.0)		
Female	5 (21.0)	2 (18.2)	3 (23.1)		
Gender non-conforming	1 (4.2)	1 (9.1)	0		
Sexual orientation				N/A	0.63
Gay, homosexual, or lesbian	16 (66.7)	7 (63.6)	9 (69.2)		
Straight or heterosexual	6 (25.0)	2 (18.2)	4 (30.8)		
Bisexual	1 (4.2)	1 (9.1)	0		
Don’t know	1 (4.2)	1 (9.1)	0		
Race				N/A	1.00
White	16 (66.7)	7 (64.0)	9 (69.2)		
Black	3 (12.5)	2 (18.2)	1 (7.7)		
American Indian	4 (16.7)	2 (18.2)	2 (15.4)		
Asian	1 (4.2)	0	1 (7.7)		
Employment status				N/A	0.92
Employed full-time	5 (20.8)	3 (27.3)	2 (15.4)		
Employed part-time	4 (16.7)	2 (18.2)	2 (15.4)		
Unemployed/disabled	12 (50.0)	5 (45.4)	7 (53.8)		
Retired	3 (12.5)	1 (9.1)	2 (15.4)		
Highest level of education				N/A	0.38
12th grade or less	2 (8.3)	0	2 (15.4)		
High school graduate/GED	3 (12.5)	1 (9.1)	2 (15.4)		
Some college/AA degree/technical school	12 (50.0)	8 (72.7)	4 (30.8)		
College graduate	3 (12.5)	1 (9.1)	2 (15.4)		
Graduate school degree	4 (16.7)	1 (9.1)	3 (23.1)		
County of birth				N/A	0.14
USA	21 (87.5)	11 (100.0)	10 (76.9)		
Other^a^	3 (12.5)	0	3 (23.1)		

*AA* Associate of Arts, *GAD*-7 general anxiety disorder 7, *GED* general educational development test, *IQR* interquartile range, *N/A* not applicable, *PHQ*-8 patient health questionnaire 8, *USA* United States of America

^a^Other countries of birth included China, Lebanon, and Brazil

While participants experiencing high and low COVID stress differed in their perceived amount of stress and their personal appraisal of their ability to cope, they did not differ in how they described the types of stressors or the relationships between stressors and corresponding strategies used to cope. Therefore, results for all participants are presented together. Most participants expressed having felt some level of anxiety and depression; however, the degree to which they experienced mental health challenges was related to their specific stressors and success with adopting positive coping strategies. Overall, among 3 main topic areas, we identified multiple themes as participants described their experiences, which are further described in the text below. Table 2 provides additional support for each theme.

**Table 2:** Illustrative quotes corresponding to pandemic-related stressors, personal buffers, and coping strategies

Topic 1. Pandemic-related stressors
Fear of COVID-19 acquisition: *“There’s still that little element, about 5%, that thinks “is this it, is this what’s going to take me out?” Here I am surviving 35 years of HIV, and COVID comes in and within a week takes me out. So there is, that’s still in the back of your mind whenever I go out, so even to doctor’s appointments, to the dentist, and QFC [local grocery store], I’m a little nervous, especially if somebody gets on the streetcar…if somebody gets on the streetcar and is not wearing a mask, one time I got off and waited for the next streetcar, so.”*—American Indian/Alaska Native man, late 60s, high COVID stress
Impact of social distancing guidelines on inter-personal relationships: *“I am a very social person. So, not being able to get out or do anything is really damping my spirit…I’m the person that my friends and family tend to call when they want that hug or when they need that good cry, and we haven’t been able to do that. So, I spend a lot of my time on video calls, zoom calls, and things like that, trying to keep the connection open, but this has been very hard, you know, not seeing my nephew has been very, very, very hard.”*—Black woman, late 30s, high COVID stress
Direct experiences with COVID-19 illness: *“Yeah, when my husband’s brother was in the hospital, he was hospitalized for like, two months. We were praying and like, we were worried. Because we thought he was gonna die, and well, thank God he didn’t. But we were anxious and very sad.”*—White man, late 20s, high COVID stress
Uncertainty in pandemic response management: *“I think probably about three or four months in, I had a little bit of a freakout. Just because there didn’t seem to be a plan in place. You know, we could see cases going up, we could see the hospitals filling up, we could see, you know, this was during that, and there was no plan. There was no plan for testing, there was no plan for anything. And I had a little bit of a moment, then. You know, but, and I think regularly, anytime I had to listen to Trump talk, I was, you know, I felt my level of anxiety and anger going up. And then when I heard the Woodward tapes of Trump, I was infuriated… So I guess, as far as like, medically, I haven’t had a lot of freak outs, maybe one or two, but politically and information wise, that’s probably been where the majority of my negative feelings have come from.”*—White man, early 50s, high COVID stress
Topic 2. Personal “buffers” or protective factors
Stable income and housing: *“We haven’t had any stress because my daughter’s still working and she makes good money. And we’re in low income housing. So that helps and…if we didn’t have any income, our rent would only be $50 at the minimum, so we would have been fine.”*—White woman, early 60s, low COVID stress
Economic independence: *“You know, I’ve been ridiculously fortunate. I mean, my business is doing better than before. My housing has gotten better. I’m healthier than I was a year ago. I have a great relationship…I already worked at home. I own my own business and worked from home or from coffee shops. So now, I just work from home using Zoom.”*—White man, mid-50s, low COVID stress
Pandemic relief: *“There’s been some interesting things that have come across that have been helpful, like the stimulus packages, and like, they have, DSHS has something called P-EBT [pandemic electronic benefits transfer]. So, I have two children who are in grade school, and they are on reduced lunch. And so we get paid in food stamps for every day they should have been in school getting reduced lunch.”*—Black gender-queer/gender non-conforming, early 30s, low COVID stress
Obtaining information: *“I think the worry has changed. In the early days, I was a lot more stressed. And worried. Because of lack of information, or that nobody really knew details about it as much as they do now. So, I feel like now I’m more prepared to be able to protect myself better and make more educated choices.”*—White woman, early 40s, low COVID stress
Topic 3. Coping strategies
Adaptive strategies
Cognitive coping: *“I mean, I take a couple of deep breaths and get aware. Like, okay,…get really tuned into what’s really in front of me… What’s really happening here?…As opposed to what I’m afraid of, or what some perception is. And often it’s like, ‘Oh, actually, I started coughing a little bit. I’ve been sneezing. It feels like I have a cold. I will go get tested. And I don’t have a result yet. And there’s nothing I can do about it until I know and I’ll know, one way or another, and if it is COVID, I will be able to accept…I think that’s how I usually deal with things like that. It’s like, okay, what’s true, and what’s made up? And what’s unknown? And if it is COVID, then [what] are the things for me to do?”*—White man, mid-50s, low COVID stress
Physical exercise: *“My husband and I decided that instead of just numbing ourselves by eating whatever treat we wanted, or, by having wine in the evening while we watch TV…we decided, this is ridiculous, we’ve had enough…it’s been really nice, because we’ve had a little bit more socialization in little bite size interactions at the gym. But it’s all still very what we feel is controlled within reason…And losing my COVID weight, and now I’m chipping away at the baby weight that I didn’t lose. It feels really good to be able to focus on something other than COVID.”*—White woman, late 30s, high COVID stress
Helping others: *“I would call them and tell them, if they need anything, let me know, I can like drop it off in front of their front door, if they need to. You know, I’m obviously not gonna go over there and give them a hug. But, you know, I wanted to make sure they knew that they weren’t alone…So, I would just offer that up to them.”*—White man, mid-40s, low COVID stress
Accessing social support: *“I still spend a lot of time on the telephone…I spend a lot of time having long conversations with people, especially those nearest and dearest, you know…And it’s just kind of that thing that sometimes that call out of the blue, that interaction you weren’t planning for is the thing that really lifts your spirits.”*—White man, early 50s, high COVID stress
Engaging in meaningful activities: *“Well, there’s not really much that I can do. But I pretty much tried to divert from baking and go more just like cooking, because a lot of baked goods have a lot of carbs and sugars, and I’m trying to get more into the proteins and the grains. So now like, I guess, like having to make that gravitation is giving me something to do. And pretty much just bettering my life is the only thing I’ve been trying do during this pandemic.”*—Black man, early 30s, low COVID stress
Maladaptive strategies (when in excess)
Harmful or hazardous alcohol use: *“[W]hen COVID started, it took away my new job, and then it closed down my gym, and then it closed down all the trails that I was hiking at. So, it took away not only my job, but took away everything that I could do for fun. So. I had already drank a lot. So COVID turned drinking into a full-time hobby, which was not healthy for me… Not seeing my boyfriend for about half a year, that was really hard.”*—*White man, early 30s, low COVID stress*
Compulsive behaviors: *“I felt myself sort of compensating by becoming overly, what’s the word I want to use, compulsive about certain things. It hasn’t been a detriment…I’ve probably been gambling more than I should.”*—American Indian/Alaska Native man, late 50s, low COVID stress
Anxiety and harmful substance use: *“I actually have one that I need to go do, speaking of lab appointments…I have a lot going on, besides the HIV. So, I have to go every two months. But, you know, it’s sad to say this, when I do go, I literally have to take an edible to relax myself. That’s how nervous and anxious I am going into the hospital.”*—Black woman, late 30s, high COVID stress
Increased smoking: *“The only time it got really bad, where I did smoke a pack, was that 24-h period where we had to wait for our [COVID-19] results.”*—American Indian man, late 60s, high COVID stress

### Topic 1. Pandemic-Related Stressors

The COVID-19 pandemic brought about a range of acute and chronic stressors, including exposure events, social isolation, and disruptions in routine services.

#### Exposure Events and Symptoms Compatible with COVID-19 Acted as Powerful Short-Term Stressors

On the individual level, common pandemic-related stressors included exposure events, onset of symptoms compatible with COVID-19, and COVID-19 illness. Many participants described being fearful of COVID-19, often stemming from their personal experiences living with a chronic viral infection and the uncertainty of how COVID-19 could affect their health. Most participants reported strictly adhering to their antiretroviral regimens even before the pandemic began; as a result, these participants did not cite non-adherence as a personal risk factor for contracting COVID-19 and developing severe complications. Rather, their living and working environments, as well as any comorbidities they had, more closely informed their personal perceptions of risk.

For most participants, COVID-19-related fear and stress were manageable on a day-to-day basis, provided they could adhere to social distancing, practice regular hygiene routines, and limit visiting crowded public spaces (e.g., grocery stores, public transportation). When outbreaks occurred in their living or working environments, or when participants experienced flu-like symptoms, their worry and stress intensified. Symptoms led to heightened stress even among participants who perceived their overall risk of severe COVID-19 to be low. After testing negative, participants often maintained or increased adherence to social distancing.

Many who perceived high risk for mortality due to COVID-19 cited HIV as an underlying heath condition that increased their risk. Highly stressed participants reported anxiety, with “tipping points” that felt beyond control due to constant worry and fear.

*“I mean, I’m probably like 80- or 90-pounds overweight, and the smoking, and then who knows what it would really do with the HIV?...Like I said, on a daily basis, it doesn’t scare me. But whenever I’ve had to go in for a test, I mean, that’s when all those thoughts are going on. It’s like, ‘Oh, my God, I’ll probably die if I get it.’ And then like right now, like today, with the ICU so overloaded and stuff, I wonder if they had to triage me, where they would put me, right? Am I just at more of a risk of dying, so they’re not going to treat me…?”*—White man, mid-40s, high COVID stress

*“You know every cough, every sniffle, every itch in your nose or itch in your throat was perceived as I have COVID. And even though we weren’t going anywhere, and we weren’t socializing with anyone, it was just that initial fear and panic... I really had a hard time with the anxiety and the depression about that. And, you know, it was a super huge trigger from an already existing gaping wound of “When am I going to die?”*—White woman, late 30s, high COVID stress

To cope with heightened worry and manage fears of contracting COVID-19, some participants developed compulsive behaviors.

*“I started making sure that I wiped down everything with Clorox wipes that came into the house as much as possible or washed it with soap and water. Everything. Fruits and vegetables, boxed goods, canned goods, everything, everything. I started washing it and sterilizing things as much as possible, and I’ve carried that on ever since.”*—White man, early 50s, high COVID stress

When asked if they knew PWH in their communities who had been diagnosed with COVID-19, many participants said ‘no’; in fact, many were surprised that the pandemic had not impacted PWH as much as they had initially anticipated. Despite not knowing many who had fallen ill with COVID-19, several participants feared contracting COVID-19 from routine clinic visits.

*“I try as much as I can not to go [to the hospital], because even if there is a protocol and we are taking as much as we can as precaution…you’re gonna sit where other people sit and breathe, and you’re in the same huge room. In my mind, even if you’re wearing a mask in the room, you can catch COVID in some way, in a small percentage. It’s not a 0% risk.”*—White man, late 20s, high COVID stress

Of the 24 study IDI participants, only four were diagnosed with COVID-19 prior to their interview. As such, most participants did not experience stress related to actual illness, treatment, or long-term complications. For the four participants who contracted COVID-19, all recovered (two after hospitalization) but expressed fears of contracting it again.

#### Social Distancing and Concern for Others Were Sources of Chronic Stress, Regardless of Fears about Contracting COVID-19

Nearly all participants expressed their support for, and followed, social distancing.

*“I don’t see anybody. No. You can’t. Because that’s congregating. No friends, nobody to see. No, no. Even I don’t like it. This is not about like or not. That’s a life and death. You either observe the social distancing and quarantine or you don’t. Do you have a choice to die? Go dig a grave, I would say. And I told people, too, please do not invite each other. You invite me, I’m going to report you because you’re congregating.”*—Asian man, mid-60s, high COVID stress

However, for many, adhering to social distancing led to isolation and loneliness. This distress stemmed from several sources: reduced face-to-face interaction with family, limited means of supporting loved ones who were struggling, and few opportunities for community gatherings and group-based activities.

Limitations in one’s ability to provide support to family members and friends who were unwell was commonly brought up. For some, this element of social distancing was extremely painful and led to sadness and helplessness. Anxiety and depressive symptoms intensified when family or close friends experienced severe COVID-19 symptoms.

In addition to feeling lonely, sad, or “down” due to being unable to engage in certain activities or social gatherings, many participants felt their families and communities were not taking the pandemic and social distancing guidelines as seriously as they ought to, which brought about feelings of anger and frustration. Several participants expressed concern over the political climate and the disproportionate impact of the pandemic on racial and ethnic minority groups.

#### Changes in Insurance and Disruptions in Medical, Dental and Social Services were Common Challenges

Overall, participants reported few challenges accessing COVID-19 testing and treatment or STI testing and treatment; however, many reported a wide variety of system navigation challenges related to changes in insurance and accessing unemployment benefits if needed. Participants also described challenges scheduling medical and dental appointments. A few also reported feeling that their health needs and concerns were invalidated by clinic and social service staff.

*“Imagine that my social worker, my case manager was yelling and screaming at me, just because I have more appointments that I have to go to every week that I wanted to get some more help for bus tickets…And they refuse me for this, that and those services…That is extremely stressful.”* –Asian man, mid-60s, high COVID stress

### Topic 2. Personal “Buffers” That acted as Protective Factors

Participants who perceived their risk of contracting COVID-19 to be largely based on personal behavior, and therefore mostly in their control, described feeling safer as they learned more about COVID-19. Along these lines, participants described a range of protective factors that helped ease COVID-19 related stresses.

#### Housing and Financial Security were Essential Personal Buffers

Only a few participants expressed concern about the possibility of being evicted from their homes. These participants had lost their jobs early in the pandemic and remained unemployed for several months. Despite participants eventually finding other jobs, these experiences had a substantial impact on their finances and personal well-being. While many other participants lived in low-income housing, none were homeless at the time of their interview, and housing and finances were not major sources of stress for them. Numerous participants described their homes as a refuge, and some felt this helped protect them from COVID-19 exposures.

*“I’m lucky to be living in a place that even though my contract at [employer] ended, I don’t need to worry about being homeless. I don’t need to worry about going to food banks and being at an extra risk. I’m really blessed to be in a stable environment.”*—White woman, early 40s, low COVID stress

One participant described how his low-income housing facility not only provided on-site COVID-19 testing, but also provided food and other forms of support for those experiencing symptoms compatible with COVID-19. Importantly, many participants who were retired, received social security/disability benefits or had lost their jobs, had spouses or other family members who provided financial assistance as needed. This added layer of financial security enabled participants to purchase food, pay bills (e.g., phone, internet, TV), and buy other necessities. Other factors that enhanced quality of life included employment (especially when the work environment was perceived as safe), home delivery of groceries or medications, and COVID-19 financial relief, among others.

#### Access to Technology Improved the Ability to Maintain Social Connections

For many, technology was the primary means of maintaining social connection early in the pandemic. Having a phone or computer and internet access, were also critical for participants who were anxious about going to medical facilities and preferred to use telehealth services. Notably, though engagement in virtual mental health services was mixed, participants who attended virtual counseling sessions often felt they benefited from valuable coping tools or practices.

*“I have a really fantastic therapist that I see once a week online. And I feel so fortunate. So, I have a great thing with her, and she’s really helpful for me…it’s one of those things where, after going to therapy for a long time, you get a really good toolbox of tools that you can use emotionally.”*—White woman, late 30s, high COVID stress

### Topic 3. Coping Strategies

Participants employed adaptive and maladaptive coping strategies to alleviate pandemic-related stress.

#### Cognitive Coping Techniques, Physical Exercise, Social Support, and Engaging in Meaningful Activities at Home were Helpful in Alleviating Stress and Building Resilience

Participants frequently drew parallels between when they were first diagnosed with HIV and the COVID-19 pandemic. Most had developed an array of techniques to help them manage their physical and mental health based on years of experience coping with HIV, in addition to other chronic conditions. These preexisting strategies allowed them to feel more prepared for coping with the COVID-19 pandemic.

Cognitive coping techniques and emotion-focused activities (e.g., positive reframing, meditation, mindfulness) were particularly useful for stressors perceived to be out of one’s personal control. For example, after experiencing symptoms and while awaiting test results, cognitive coping techniques helped participants remain calm.

*“It always goes back to HIV. With those of us that have HIV, it’s that same anxiety you feel and fears that you feel when you’ve taken the test that, back then we had to wait seven days, and fortunately with this, we only had to wait 24 hours. And so, there was a lot of high anxiety, but I just started doing my self-meditation and thinking that, you know, there’s no sense in stressing about it until you hear.”*—American Indian/Alaska Native man, late 60s, high COVID stress

Emotion-focused strategies were helpful for coping with the open-ended nature of the pandemic. For example, many participants spoke about the pandemic’s early months as being the most difficult, especially as there was limited information on how long it would last. Meditation and mindfulness were frequently cited by participants as helping them practice gratitude and positive thinking over time.

For some participants, physical activity was one of the most impactful coping strategies for combatting prolonged stress and building resilience. Participants who exercised often reported improvements in depressive and anxiety symptoms. For many, exercise provided more than physical health benefits, serving as a distraction and means of keeping busy. Participants who worked, and especially those who worked outside their homes, also expressed less social distancing stress compared to those who spent much of their time at home. For participants who did not regularly exercise, common reasons provided centered around (1) not feeling safe exercising in public spaces, or (2) having underlying health conditions that made engaging in prolonged physical activity difficult.

*“I was a member in a gym, and I canceled it. And then I was trying to workout outside the house. But like no one, like even if I’m running or doing some exercise outside the house, in the park or wherever, people don’t wear a mask. And even working out or running with the mask is something that I honestly don’t like. And I feel like I can’t breathe. So, I stopped doing that.”*—White man, late 20s, high COVID stress

Participants found ways to stay connected with friends, family or neighbors via the following forms of social support: emotional (e.g., physical or virtual presence), instrumental (e.g., food deliveries, financial assistance), and informational (e.g., sharing COVID-19 information and advice), which helped alleviate depressive and anxiety symptoms.

Finally, many participants also engaged in meaningful activities at home, including home improvement projects and hobbies such as making art, reading, watching TV, and cooking new recipes. Many such activities served as important opportunities for self-reflection, personal growth and self-care.

*“I think, in a weird way, this isolation at home alone has given me a really long time to recover on my own in a way that has helped me specifically, gathering my own thoughts and look back on things, and, what’s the word I’m looking for? A chance to review things long term and come to some really important life decisions and choices that have helped me in a tremendous way.”*—White man, early 50s, high COVID stress

#### Job Loss and Social Distancing Stress Increased Depressive Symptoms and Exacerbated Preexisting Tendencies to Engage in Maladaptive Coping

Participants reported feeling down for a number of reasons, including job loss, inability to socialize in person or take vacations, and loss of loved ones. Some participants with a history of depression reported recurrent depressive symptoms and restarting therapy.

*“I lost my job because of COVID. And so, you know, I was really depressed through the whole summer, which was part of the job loss, but I mean, just lonely. You know? I’m not a huge social person, like, I don’t go out to bars and stuff. But I usually take a vacation. I go somewhere every summer and during the winter, I definitely go somewhere sunny to fight my seasonal depression. And so now, that’s not happening. I’m just sort of worried about my grandma a lot…I went on a second pill for my depression.”*—White man, mid-40s, high COVID stress

While most participants had either been sober for many years or had not changed their consumption of alcohol, tobacco, marijuana, or illicit substances during the pandemic, some participants who engaged in maladaptive coping strategies prior to the pandemic (e.g., harmful or hazardous alcohol consumption, harmful substance use, excessive gambling) noted that pandemic-related stressors increased their frequency of engaging in these unhealthy habits. For some participants, a combination of stressors acting together contributed to unhealthy consumption.

*“So, I drink two glasses of wine per night to keep my blood sugar down…Before though, when the pandemic first started, it was like two bottles of wine. You know, you can get really cheap wine nowadays. And I feel like that was because of the pandemic, like I don’t have to be at work tomorrow, and well, I might as well get drunk. It was kind of like, I feel like that’s only because of the pandemic. I felt like it’s okay to do that.”*—Black man, early 30s, low COVID stress

Fortunately, several participants who engaged in maladaptive coping strategies were connected to, or increased participation in, mental health counseling, Alcoholics Anonymous, or other behavioral health services.

### Updated Stress-Coping Model

**Fig. 1:**
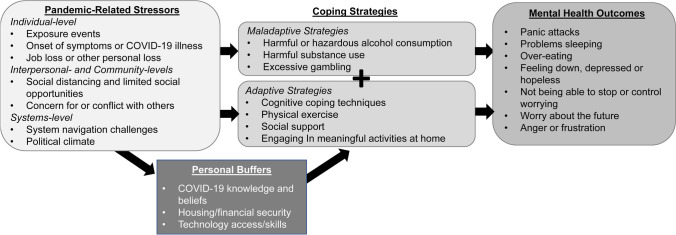
Updated COVID-19 stress-coping model

Lived experiences of participants were used to expand our initial stress-coping model to accommodate the complex interactions between individual stressors, personal buffers, coping strategies, and mental health outcomes among PWH during the COVID-19 pandemic (Fig. 1). Our adapted model includes the following stressors: individual-level stressors (e.g., anxiety about acquiring COVID-19), inter-personal level stressors (e.g., social distancing stress or conflict with family or friends), community-level stressors (e.g., limited social opportunities) and systems-level stressors (e.g., system navigation challenges, political climate). These stressors were either short-term or longer term, and they ebbed and flowed with the pandemic. Exposure events and symptoms compatible with COVID-19 usually led to short-term stress, while most participants described prolonged stress stemming from social distancing and limited opportunities for interpersonal support. Participants who experienced multiple stressors often engaged in maladaptive coping, especially after job loss or some other adverse event. Personal buffers such as stable housing and job security protected those who had these advantages. Regardless of personal buffers, adaptive coping strategies helped most participants cope with these stressors in positive ways and reduced the negative impacts of COVID-19 on mental health outcomes. When navigating systems level challenges, healthcare providers played a critical role in supporting PWH, by providing continuity in care as well as referrals to mental health and social services.

## Discussion

Our qualitative evaluation of lived experiences of PWH in Western Washington suggests that a complex interplay of stressors at the individual, interpersonal, community and systems levels affected PWH experiences coping with the COVID-19 pandemic. Participants identified both adaptive and maladaptive coping strategies used to alleviate stressors and reduce impacts on mental health outcomes. Personal buffers, including knowledge and beliefs about COVID-19, job security, and access to technology, helped promote adaptive coping responses and reduce negative mental health impacts. Building upon previous stress-coping research, participant experiences were used to expand an initial stress-coping model to characterize the interactions between identified stressors, personal buffers, coping strategies and mental health impacts among this population. To our knowledge, this is one of the first research studies to provide a stress-coping model that articulates common mechanisms through which the COVID-19 pandemic impacted the mental health of PWH. While our model is similar to the ecological model published by Cowan et al., illustrating individual, network, community, and structural features of the pandemic impacting the health of individuals with opioid use disorder [23], this and other models published in the literature do not include successful coping strategies or other factors which promote resilience [24].

Our findings align with other research studies suggesting that problem-focused and emotion-focused coping activities that improve mood, such as finding gratitude via mindfulness, exercising, and having a supportive social network, promoted well-being during the COVID-19 pandemic [25–29]. Similar to other studies, we found that having access to technology was an invaluable resource for maintaining social connections and giving and receiving social support during the pandemic [29]. While these strategies are not necessarily different or novel from those shown to be effective for coping with HIV prior to the COVID-19 pandemic [16–18], these findings underscore the importance of developing and maintaining skills and behaviors to effectively manage short and longer-term stressors. Importantly, results from this study are also supported by previous research which suggests that prior experience coping with HIV and other chronic conditions helped some PWH feel more prepared to cope with pandemic-related stressors [30, 31].

Although some participants remained physically active during the pandemic, many participants did not describe physical activity as part of their daily routine. This finding is supported by a recent study conducted by Wion et al., which found that aspects of HIV self-management that were made the most difficult by the COVID-19 pandemic were “the ability to exercise, ability to manage affective symptoms, and ability to maintain social support networks” [32]. This is cause for concern, as a growing body of literature suggests that exercise improves depressive and anxiety symptoms, increases quality of life, and promotes treatment adherence among PWH [32–34]. Equitable access to physical activity should be further explored for PWH who have limited outdoor recreational opportunities or don’t feel safe exercising outside of their homes. Likewise, additional research is needed to evaluate the clinical effectiveness of virtual physical therapy sessions, as well as patient barriers to using these services [35–37]. Addressing these gaps in knowledge will be particularly important for individuals who have physical limitations in their ability to exercise.

It is important to note that the COVID stress scores used for purposive sampling in this study were not a proxy for having experienced stressful events such as job loss or traumatizing events such as domestic violence. In fact, some participants who were categorized as having “low COVID stress” had experienced many of these difficulties, but these experiences did not necessarily impact their perceptions of stress related to the COVID-19 pandemic specifically. The level of perceived COVID-19 stress for each participant was dependent on individual-level and other contextual factors, and limited characterization and comparison of different stressors or coping strategies by COVID-19 stress score category.

While all participants described access to COVID-19 testing, PWH described challenges accessing social services, such as insurance and unemployment benefits, and scheduling routine medical and dental appointments. Thus, our findings suggest that an evaluation of how changing clinic and social service policies impacted patients’ sense of safety and service access during the pandemic would be beneficial. Community advisory boards and other avenues for patient feedback will be critically important in developing patient-centered solutions that are equitable and inclusive, and in improving patient–provider relationships in future COVID-19 waves or future pandemics.

We acknowledge several limitations to this study. First, our data reflect the experiences of individuals who had access to e-mail and were willing to be contacted for an interview, responded to our invitation, and consented to use either online Zoom or telephone-to-Zoom to participate. Second, we note that the median age of survey participants was lower than that of HIV patient registry participants, at 47 versus 52, which may be due to differences in technology access or skills. Third, the response rate to our interview invitations was relatively low, at 41% overall. This sampling bias was, in part, unavoidable due to social distancing restrictions, as in-person recruitment and data collection were not possible during the study period. As a result of this limitation, we likely under-sampled individuals who experienced homelessness or extreme mental health and substance use challenges, and our results may have limited generalizability to more vulnerable populations. However, our study draws on the experiences of individuals who reflect the socio-demographic profile of many HIV clinic patients in Western Washington, in terms of age, race/ethnicity, gender identity, and sexual orientation. Fourth, the seven-item COVID stress scale used in this study was created at a time when validated tools were not available, and it has not been formally validated. Finally, of the 24 participants, only four had been diagnosed with COVID-19 prior to being interviewed. As such, we cannot provide generalizable findings regarding experiences with illness, stigma, or other adverse outcomes for those diagnosed with COVID-19.

### Conclusion

In summary, we collected interview data from 24 individuals who receive their HIV care at UW clinics in Western Washington. Cognitive techniques, physical activity, and social support appeared to be the most impactful coping strategies for participants during the first year of the pandemic. Although COVID-19 vaccines and other treatments are now available and social distancing guidelines have been lifted, understanding how PWH experienced stressors and coped during the COVID-19 pandemic can help healthcare providers connect with their patients, address mental health needs and support adaptive coping strategies during future public health emergencies.

### Supplementary Information

Below is the link to the electronic supplementary material.

Supplementary file1 (DOCX 33 KB)

## Data Availability

Data and material will be available upon request. Please contact the corresponding author.

## Code Availability

Code will be available upon request. Please contact the corresponding author.
