# Supplementary material for: Impact of the COVID-19 Pandemic on Persons Living with HIV in Western Washington: Examining Lived Experiences of Social Distancing Stress, Personal Buffers, and Mental Health
Source: AIDS Behav. 2024 Mar 16;28(6):1822–33. doi: 10.1007/s10461-024-04273-7 (PMC11161538; doi:10.1007/s10461-024-04273-7)
Supplement: Supplementary file 1 — Supplementary file1 (DOCX 33 KB) [file 10461_2024_4273_MOESM1_ESM.docx]

**Impact of the COVID-19 Pandemic on Persons Living with HIV in Western Washington**

**Individual Interview Question Guide**

**Interviewer Instructions:**

The following is a guide. Try to ask all the questions below in the order given, but it is more important to maintain the flow of discussion. Suggested probes have been included. Start with the following introductory script.

NOTE: PARTICIPANTS MAY BE EXPERIENCING LOSS AND GRIEF DURING THIS PERIOD. PLEASE MAKE REFERRALS AND OFFER SUPPORT AT THE END OF THE INTERVIEW AS APPROPRIATE.

******************************************************************************************************************************

**Before turning on the recorder, start with the following introductory script:**

*Hi, my name is ____________. Thank you for agreeing to participate in an interview today. As you may know, the COVID-19 pandemic has really changed how things are happening in our society. Social distancing guidelines have changed how we can get health care and services, whether schools are open, whether stores are open, what jobs are available, and where people can meet and how they can each other. It has also made many people really sick, and they might have been hospitalized, or even died from COVID-19, or another illness.*

*During our discussion today, we are interested in hearing about your experiences during the COVID-19 pandemic, including how it has affected your health and the health of those you know. We are also interested in hearing how COVID-19 has affected your HIV care, including how you access medications and what clinic appointments are like.*

*During our discussion today, I will ask you questions that you are free to answer in any way you wish. Feel free to elaborate on any of your points. If a question is unclear to you, please feel free to ask me to explain it.*

*I would like to record the discussion so I don’t miss anything that you say. I will not include your full name on any documents or in the recording. Your answers will be kept confidential, which means we will keep what you say private from others. Is it okay if I record our discussion?* [Wait for the participant to give verbal consent to recording]

*Before we start I would like to remind you that there are no wrong answers during our discussion. We are interested in knowing what you think, so please feel free to be open and share your point of view. We hope you can help us understand how the COVID-19 pandemic has affected you and those you know, and how we can improve the care and services offered to you in the future during a similar situation with COVID-19, or another similar situation.*

*If you want to stop the discussion at any time, just let me know. Do you have any questions for me before we get started? [Wait for participant to respond – answer any questions they have]*

*I am turning on the recorder now.*

******************************************************************************************************************************

**Before beginning interview questions, please read the following script for the recording:**

*Today is [day of week], [month, day, year] and it is now [time of day]. This is interview [ID number] conducted at [discussion location].*

**You are now ready to begin asking the questions outlined below.**

1. First, I’d like to learn a little more about what you know about COVID-19.

- What have you heard about the new coronavirus, also called COVID-19 or SARS-CoV-2?
- Who or where do you get your information from?
  - What do you think about the accuracy of what you have heard?
  - What sources of information do you trust?
- Have you received and/or asked for any guidance from any of your health care providers about the new corona virus? Did you get enough information? Why or why not?

1. Now, I’d like to talk with you about your beliefs about your risk of getting COVID-19.
   - - - Do you think people living with HIV are more at risk or less at risk for the new corona virus than people not living with HIV?
       - How concerned are you about your getting (that is, contracting) the new corona virus?
         - Why do you feel that way?
         - Has your concern changed over time? Why or why not?
       - Did you ever have any symptoms or situations that made you think that you might have had COVID-19? Did anyone you know have COVID-19?
         - Please tell me about your experience (or the experience of someone you know) having COVID-19?
         - What was your experience with symptoms?
         - What was it like to get tested for COVID-19?
         - How did you or healthcare providers manage your (your loved one’s) illness?
         - What could healthcare workers have done better to improve your experience?
         - Have you (or someone you know) experienced any longer lasting health effects from COVID-19?
         - Are you scared about getting COVID-19 again? Why or why not?
2. Now, I’d like to talk with you about your experiences with social distancing and how that has influenced what your life is like.
   - - - Overall, how has your quality of life changed since coronavirus? Probes: Do you feel more or less socially connected? More or less engaged in activities? More or less bored?
       - Have you experienced depression, sadness, or grief?
       - Have you experienced anxiety or additional stress?
       - Have you experienced any other major changes in mood?
       - How do these experiences or feelings affect your ability to manage your mental health?
     - How has the corona virus and/or social distancing affected other parts of your life?
       - - How has your job or how you get money changed?
         - How has your ability to see friends and family changed?
       - How has your sexual behavior, sexual health, or relationships changed?
         - How has your ability to get food/stay housed changed?
         - How has your substance use patterns or thoughts about substance use, including alcohol, marijuana, other drugs, and tobacco changed?
       - What has been the most challenging part of the social distancing guidelines?
       - What have you done to stay connected with friends and family? How do you feel that’s been working for you?
       - What else have you done to cope during corona virus?
3. Next I’d like to hear more about how COVID-19 has affected your desire or ability to access HIV care.
   - - - What was working well for HIV care before COVID-19?
       - What were some of the challenges with your HIV care before COVID-19?
       - How has your HIV care changed since COVID-19?
         - How has COVID-19 changed how you access medications? What has been hard/easy about these changes?
         - How has COVID-19 changed your adherence to medications? Why?
         - What do your clinic visits look like during COVID-19? Have you used telemedicine? Why/why not? What did you like/not like about that?
         - How has getting to clinic changed since COVID-19? How have you liked or not liked those changes?
         - What has been different about your ability to get social services? What about insurance? What about counseling and support? What has been working well with these changes? What has not been working well?
       - How has it affected your desire or ability to access other types of health care for conditions other than HIV?
       - What do you and members of your community need to manage life during the corona virus outbreak that you are not getting?

As we finish taking today, **i**s there anything else you think I should know about how the coronavirus is affecting how you manage your HIV-related health care and medications?

Is there anything else we should know about how the coronavirus is affecting any other aspect of your life? Your neighborhood? The community of persons living with HIV?

That is all of the questions I have for you today. Thank you for your time. If you do not have any further questions or comments, I will now turn off the recorder*.*
